# In vivo biocompatibility evaluation of 3D-printed nickel–titanium fabricated by selective laser melting

**DOI:** 10.1007/s10856-022-06641-y

**Published:** 2022-01-21

**Authors:** Hendrik Naujokat, Ali Ihsan Gökkaya, Yahya Açil, Klaas Loger, Tim Klüter, Sabine Fuchs, Jörg Wiltfang

**Affiliations:** 1 Department of Oral and Maxillofacial Surgery, University Hospital of Schleswig-Holstein, Campus Kiel, Arnold-Heller-Straße 3, 24105 Kiel, Germany; 2 Department of Trauma Surgery, University Hospital of Schleswig-Holstein, Campus Kiel, Arnold-Heller-Straße 3, 24105 Kiel, Germany

## Abstract

Nickel–titanium (NiTi) belongs to the group of shape-memory alloys (SMAs), which are characterized by flexibility and reversible deformability. Advanced techniques in 3D printing by selective laser-melting (SLM) process allow the manufacturing of complex patient-specific implants from SMAs. Osteosynthesis materials made of NiTi could be used for minimally invasive surgical approaches in oral- and maxillofacial surgery. However, the in vivo biocompatibility has not yet been fully investigated, especially in load-sharing and load-bearing implants. The aim of this study was to evaluate the in vivo biocompatibility of SLM-produced NiTi for intraosseous and subperiosteal applications. Test specimens were implanted into the frontonasal bone of ten miniature pigs. To assess peri-implant bone metabolism, fluorescent dye was administered after 2, 4, 6, 10, 12, and 14 weeks intraperitoneally. Specimens and the surrounding tissues were harvested after 8 and 16 weeks for histological analysis. While the NiTi implants presented a higher bone-to-implant contact ratio (BIC) after 8 than after 16 weeks (43.3 vs. 40.3%), the titanium implants had a significantly higher BIC after 16 weeks (33.6 vs. 67.7%). Histologically, no signs of peri-implant inflammation or foreign-body reaction were detectable. With respect to this preliminary study design, 3D-printed NiTi shows sufficient biocompatibility for intraosseous and subperiosteal implant placement.

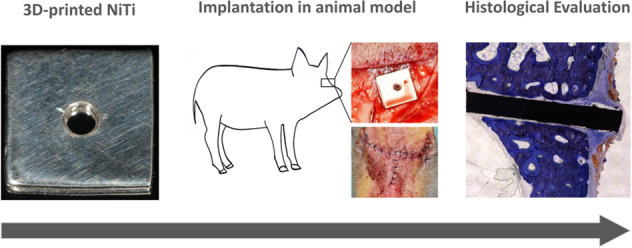

## Introduction

The use of permanent osteosynthesis and reconstruction materials is common in oral- and maxillofacial surgery [1]. Depending on the indications and anatomic site, implants of various designs and mechanical properties can be acquired on the market [2]. For example, orbital floor fractures are treated with thin meshes, mid-facial fractures with miniplates, and mandibular defects with loadbearing reconstruction plates [3, 4]. Currently, most of these implants are made of titanium or its alloys, presenting favorable biocompatibility and suitable mechanical properties [5]. In recent years, the use of patient-specific implants (PSIs) has become the standard of care for some conditions. Usually, PSIs are virtually planned and manufactured by a selective laser-melting process using well-established titanium alloys [6]. Another trend in recent years has been the development of minimally invasive and endoscopic surgical techniques. Titanium plates and meshes are rigid and nondeformable and therefore are not suitable for minimally invasive procedures.

Nickel–titanium (NiTi) belongs to the group of shape-memory alloys (SMAs), which are characterized by flexibility and reversible deformation. Due to superelastic properties, NiTi is able to regain its original macroscopic structure after deformation [7]. These mechanical properties have led to numerous indications for its use in medical implants (e.g., stents) and dental care equipment (e.g., endodontic instruments) [8]. NiTi implants are well suited to advance minimally invasive and endoscopic surgery. For example, in orbital floor reconstruction, a preformed mesh could be rolled or folded for minimally invasive insertion and then self-expanded at the targeted position. The foundation for these procedures is the patient-specific manufacturing of SMAs. Compared with conventional fabrication methods, modern additive manufacturing enables the production of highly complex NiTi implants for minimally invasive procedures. In this study, modified SLM protocols have been used for the 3D printing of NiTi [9]. However, the alloying of nickel in implants remains under discussion, as the risk of side effects might be higher than in high-purity titanium [10]. In contrast to already-established NiTi products in health and dental care, contact with different types of tissues as well as the impact of mechanical loading are factors that might influence the biocompatibility of NiTi implants. The aim of this study was the in vivo evaluation of the biocompatibility of 3D-printed NiTi implants for intraosseous and subperiosteal indications.

## Materials and methods

### Experimental animals

The animal experiments were conducted according to the European Animal Welfare Legislation and the European Directive 2010/63/EU, and they complied with the ARRIVE guidelines. The study protocol was approved by the Directorate of Food-Chain and Animal Healthcare, Hungary (Approval No. PEI/001/961-2/2013). The study included ten skeletally mature miniature pigs (Ellegaard Göttingen Minipigs A/S, Dalmose, Denmark) with an average age of 10 months and an average weight of 22–24 kg. The animals were kept under conventional conditions equipped with straw in groups of three and four with food and water ad libitum. Each animal was treated bilaterally and at two anatomical sites to achieve a total of ten specimens per treatment group (according to the sample-size estimation) and to keep the number of animals as low as possible (according to the principle of “Reduction”).

### Implants

**Fig. 1:**
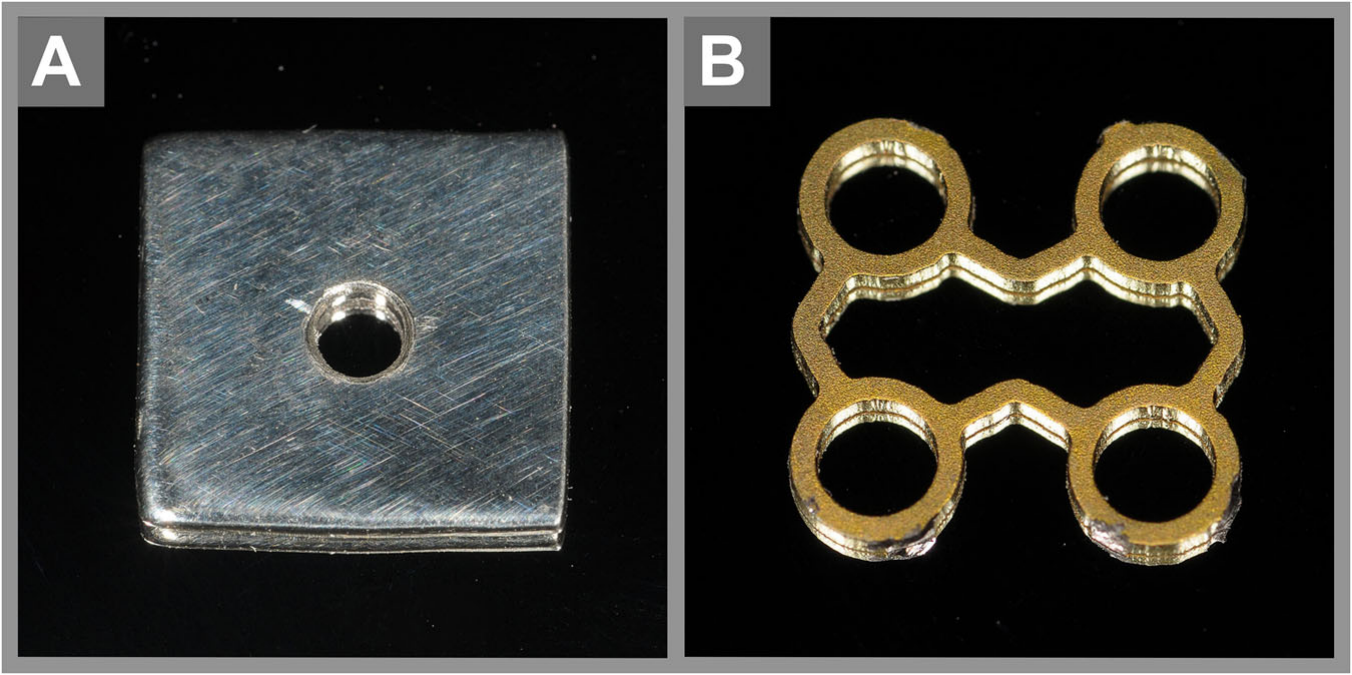
Photographs of the subperiosteal implants measuring 10 × 10 mm^2^. The NiTi implant (**A**) was manufactured with a central screw hole. The control titanium implant was cut from a commercial dynamic mesh consisting of struts and screw holes. The titanium mesh was obtained from Stryker GmbH (Dynamic Mesh 54-00647, thickness 0.6 mm, Stryker GmbH, Duisburg, Germany) (**B**)

Superelastic NiTi implants were manufactured by 3D printing using a selective laser melting process. First, sheets with a thickness of 0.2 ± 0.02 mm were printed using BB-alloy (55 weight% nickel, A_s_ (f.a.) −15 ± 10 °C, active A_f_ + 5 °C). The sheets were annealed flat at 500 °C. The surface was made oxide-free by post-annealing etching (Memry, Bethel, Connecticut, USA). The test specimens were cut via a Laser MicroJet®, a system that combines a laser and water jet to prevent thermic and mechanical damage to the material (Fraunhofer-Institut für Werkzeugmaschinen und Umformtechnik IWU, Dresden, Germany) [11]. The subperiosteal implants measured 10 × 10 mm with a central screw hole with a diameter of 1.6 mm for fixation. The intraosseous implants measured 5 × 10 mm. As a control, an established and commercially available titanium mesh was used. The mesh consisted of titanium, and due to the configuration of the struts, it was plastic-deformable (Dynamic Mesh 54-00647, 200 × 200 mm, thickness 0.6 mm, Stryker GmbH, Duisburg, Germany). The mesh was trimmed to the same dimensions as the NiTi implants above (Fig. 1).

### Surgical procedure

**Fig. 2:**
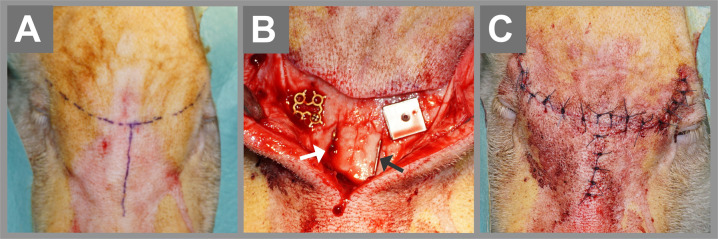
Photographs of the surgical procedure: the tip of the nose is located at the lower edge of the pictures. An arch incision was combined with a nasal extension to expose the frontal and nasal bones (**A**). A full-thickness incision was performed through the skin, subcutaneous tissue, and periosteum using a scalpel no. 15. By blunt dissection the periosteum was elevated and the bone exposed. The subperiosteal implants were adjusted on the surface of the frontal bone and fixated with one screw each (self-cutting titanium screw, diameter 1.7 mm, length 5 mm). The intraosseous implantation was performed underneath the frontonasal suture on the thickest part of the nasal bone. A longitudinal osteotomy was prepared bilaterally using a rotating diamond-coated cutting disk. Titanium was inserted on the right side (white arrow), and NiTi was inserted on the left side (black arrow in **B**). The intraosseous specimens were stable by friction forced. Wound closure was performed by a three-layer suture (**C**)

A single surgeon with an experienced team performed all operations on the animals in a standardized manner. The animals were sedated via intramuscular injection of 10 mg/kg ketamine and 1.5 mg/kg midazolam, and anesthesia was maintained by intravenous titration. The miniature pigs were placed in a prone position and prepared for aseptic surgery at the frontonasal bone. Additional local anesthesia was achieved by injection of 4 ml of 4% articaine, and the frontal and nasal bones were approached by an arched incision. At the frontal bone, the implants were adjusted on the surface for subperiosteal implantation, and a screw hole with a diameter of 1.4 mm was drilled with sterile water cooling. The implants were fixed using a self-cutting titanium screw (diameter 1.7 mm, length 5 mm, 50-17005, Stryker GmbH, Germany). The intraosseous implantation was performed underneath the frontonasal suture on the thickest part of the nasal bone. A longitudinal osteotomy was prepared bilaterally using a rotating diamond-coated cutting disk with sterile water cooling (Fig. 2). The intraosseous specimens did not need screw fixation due to the friction generated in the osteotomies. To visualize bone metabolism, 15 mg/kg Calcein green (Calcein Disodium salt, Fluka, Honeywell Int. Inc., Morristown, USA) was injected intraperitoneally after 2, 4, and 6 weeks. After 8 weeks, euthanasia was performed on 5 of the 10 animals. After 10, 12, and 14 weeks, 30 mg/kg Alizarin red (Alizarin-3-methylamine-N, N-diacetic acid dehydrate, Merck Chemicals, Darmstadt, Germany) was injected intraperitoneally. After 16 weeks, euthanasia was performed on the remaining five animals, and bone blocks with the implants as well as the surrounding tissue were stored in 4% formalin for two weeks.

### Histological analysis

The fixed specimens were dehydrated using alcohol of ascending concentrations in an embedding station (Pool of Scientific Instruments, Type 1.42.00, PSI Grünewald, Laudenbach, Germany) and embedded in methyl-methacrylate (Sigma-Aldrich, St. Louis, USA). The probes were cut with a diamond-coated saw (Cut-grinder primus Diamant, Walter Messner, Oststeinbek, Germany) perpendicular to the subperiosteal and intraosseous implants, and nondecalcified slices of 40 μm thickness were prepared (DP-U4, Struers GmbH, Willich, Germany). First, an evaluation by fluorescence microscopy was performed (Axio Observer Z1, Axio Cam and Axio Vision, Carl Zeiss Mikroskopie, Jena, Germany). Subsequently, toluidine blue staining and light microscopy were performed. The images were assessed digitally using a Leica QWin Standard (V 3.2.0 Leica Microsystems Imaging Solution, Cambridge, United Kingdom) for histomorphometric analysis. The subperiosteal specimens were evaluated by using a semiquantitative scale to quantify the bone overgrowth (0, +, + +, + + +) and peri-implant bone morphology (0, −, − −, − − −). The intraosseous specimens were evaluated by quantifying the peri-implant bone morphology with the above-mentioned semiquantitative scale and by the bone-to-implant contact ratio (BIC) at the interface.

The soft tissue that covered the osteosynthesis material was embedded in paraffin, cut into 6 μm-thick preparations, and stained with hematoxylin–eosin (Sigma-Aldrich, St. Louis, USA). For histological evaluation, the counts of macrophages, granulocytes, foreign-body cells, and lymphocytes were quantified in ten fields of view using 400-times magnification. In addition, the specimens were analyzed for tissue fibrosis and vascular ingrowth using a semiquantitative scale (− −, −, 0, +, + +). Soft tissue from the forehead harvested distant to the implants served as a negative control and was used to calibrate the scales to zero. The analyses were conducted by a researcher who was blinded to the group to which each sample belonged.

### Statistics

All data were collected and presented as the means and the standard errors of the means. Statistical calculations were performed using univariate analysis of variance (SPSS Statistics 20.0, International Business Machines Corporation, Armonk, USA). The significance level was established for a *p*-value of 0.05.

## Results

### Baseline results

The placement and fixation of the test specimens was safe and predictable. The surgical procedure and the inserted materials were well tolerated by all animals. Clinical examination revealed no signs of local inflammation, wound-healing disturbance, allergic reactions, or toxic side effects. The data of all animals were included in the evaluation.

### Peri-implant bone structure

**Fig. 3:**
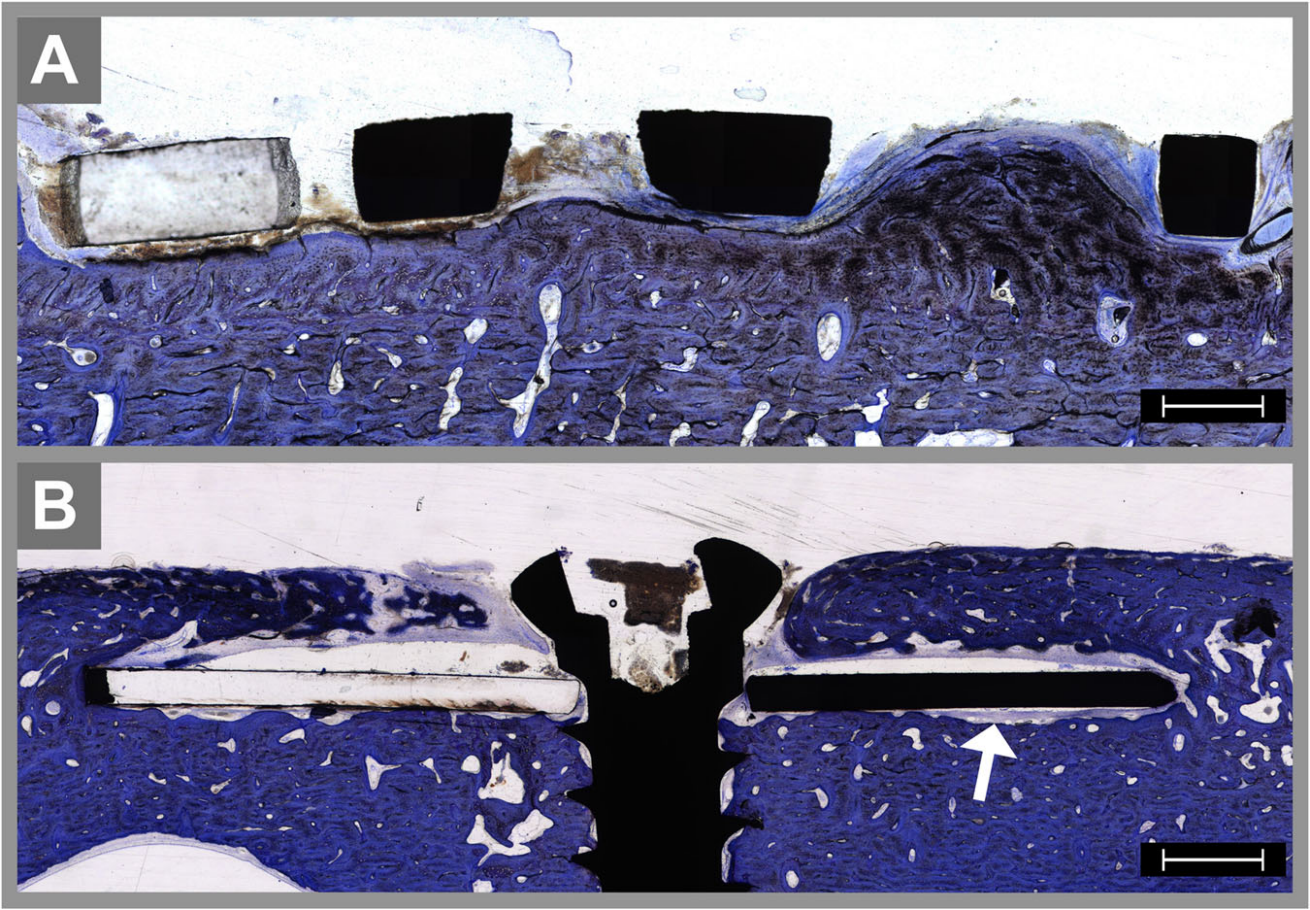
Histological specimens of subperiosteal implants after 16 weeks stained with toluidine blue. The titanium mesh had direct contact with the surface of the frontal bone and presented with some connective tissue ingrowth between the struts and little appositional bone growth at the edges of the implant (**A**). The original bone surface presents some resorption underneath the NiTi test specimen (arrow in **B**), but there is no formation of lacunae adjacent to the implant. Bone overgrowth is more extensive and results in nearly complete coverage of the implant. The scale bar is 1000 µm

The subperiosteal specimens were placed on the surface of the forehead bone underneath the periosteum. Appositional bone growth as well as surface resorption caused by the surgical procedure was plausible. However, the main histological finding was bone overgrowth at the edges of the specimens of divergent extents. After 8 weeks, the median finding in the NiTi group was appositional bone tissue that did not reach the surface of the implants, in contrast to the lack of bone overgrowth observed for the titanium specimens. Comparing the specimens after 8 and 16 weeks, increasing bone overgrowth was detected in both groups: an increase from (+) to (+ +) in the NiTi group and from (0) to (+) in the titanium group. After 16 weeks, two NiTi specimens showed complete bone overgrowth of the implant, while this was the case for just one titanium specimen (Table 1). The appositional bone presented a partially woven and a partially lamellar structure with a large number of osteoblasts. Even after 16 weeks, the density of the bone overgrowth was slightly lower than the density of the bone of the forehead (Fig. 3). The peri-implant bone tissue showed the following morphological changes: after 8 weeks, two specimens in the NiTi group showed no altered bone structure, and three showed slight resorption of the adjacent bone (median −). In the titanium group, one specimen showed no altered bone structure, two showed slight resorption of the adjacent bone, and two showed slight formations of lacunae (median −). After 16 weeks, two NiTi and only one titanium sample presented with slight formation of lacunae in the peri-implant bone (median - in both groups, Table 1).

**Table 1:** Histomorphometric evaluation of the peri-implant bone tissue

		week 8		week 16	
		NiTi (*n* = 5)	titanium (*n* = 5)	NiTi (*n* = 5)	titanium (*n* = 5)
*Subperiosteal specimens*					
Bone overgrowth	(0)	2	4	0	2
	(+)	1	1	0	1
	(+ +)	2	0	3	1
	(+ + +)	0	0	2	1
	**median**	**(+)**	**(0)**	**(+ +)**	**(+)**
peri-implant bone morphology	(0)	2	1	1	2
	(−)	3	2	2	2
	(− −)	0	2	2	1
	(− − −)	0	0	0	0
	**median**	**(−)**	**(−)**	**(−)**	**(−)**
*Intraosseous specimens*					
peri-implant bone morphology	(0)	0	0	0	3
	(−)	1	3	2	2
	(− −)	3	1	3	0
	(− − −)	1	1	0	0
	**median**	**(− −)**	**(−)**	**(− −)**	**(0)**
Bone-to-implant contact ratio	[%]	43.3	33.6	40.3	67.7
	***p*-value**		**0.57**		**0.02**

The bone overgrowth was quantified by a semiquantitative scale: (0) no bone overgrowth; (+) appositional bone tissue that does not reach the surface of the implant; (++) appositional bone that partially covers the surface; and (+++) complete bone overgrowth of the implant. The peri-implant bone morphology was quantified by a semiquantitative scale: (0) no altered bone structure; (−) slight resorption of the adjacent bone; (− −) slight formation of lacunae; and (− − −) severe formation of lacunae. The median for each group is presented in bold. After 8 weeks the difference in the BIC between the NiTi and the titanium group are nonsignificant (*p*  = 0.57). After 16 weeks the BIC was significantly higher in the titanium group (*p* = 0.02).

**Fig. 4:**
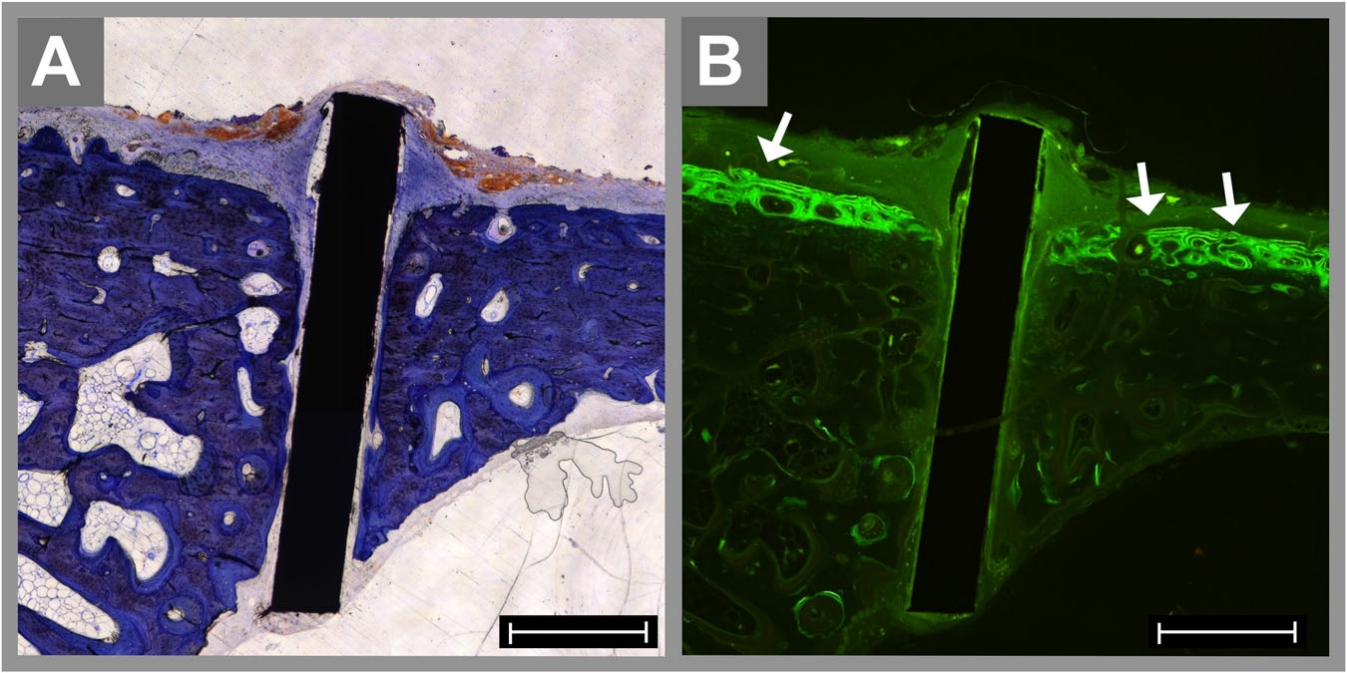
Histological specimens of an intraosseous NiTi implant after 8 weeks stained with toluidine blue (**A**) and visualized by fluorescence microscopy (**B**). The profound edge of the implant reaches the epithelium of the nasal cavity. The enrichment of green fluorescence proves the existence of newly formed bone tissue at the surface of the nasal bone (arrows in **B**); in contrast, there is nearly no fluorescence enrichment at the bone–implant interface in the osteotomy, resulting in a low bone-to-implant contact ratio. The scale bar is 1000 µm

For the intraosseous specimens, the peri-implant bone was analyzed on both sides. After 8 weeks, four NiTi specimens presented lacunae (median − −), whereas two specimens in the titanium group showed the formation of slight lacunae in the adjacent bone (median −). In cases with poor bone regeneration at the implant–bone interface, nearly no enrichment of fluorescence could be detected adjacent to the implant (Fig. 4). After 16 weeks, the morphological changes in the NiTi group did not significantly diverge from the findings after 8 weeks (median − −). In the titanium group, a decrease in the alterations of the bone tissue was detectable over time. After 16 weeks, three specimens showed no sign of alteration of the bone structure, and only two specimens showed slight resorption at the implant–bone interface (median 0).

### Histomorphometric analysis of the bone-to-implant contact

**Fig. 5:**
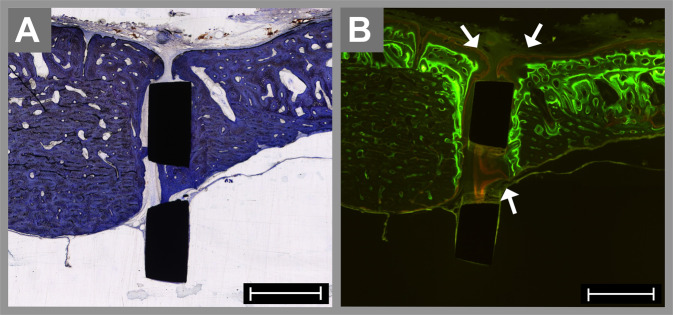
Histological specimens of an intraosseous titanium implant after 16 weeks stained with toluidine blue (**A**) and visualized by fluorescence microscopy (**B**). The osteotomy and the space between the mesh struts are not completely healed by bone tissue. However, the newly formed bone shows strong enrichment of the green fluorescence in concentric bends, and the red fluorescence is mainly located at the outer layer of the appositional bone and between the mesh struts (arrows in **B**). The scale bar is 1000 µm

Measurements of the BIC of the intraosseous and subperiosteal implants were obtained. After 8 weeks, the BIC was 43.3% in the NiTi group and 33.6% in the titanium group, which was a nonsignificant difference (*p* = 0.57). After 16 weeks, the BIC was 40.3% for the NiTi specimens and 67.7% for the titanium group, which was significantly higher (*p* = 0.02). In accordance with these histomorphometric findings, most of the titanium specimens show increased enrichment of fluorescence. The green fluorescence indicated bone formation 2–6 weeks after implantation, and red fluorescence, 10–14 weeks after insertion. In the titanium group, new bone formation at the bone–implant interface was also detectable between the mesh struts (arrows in Fig. 5).

### Peri-implant soft tissue

**Fig. 6:**
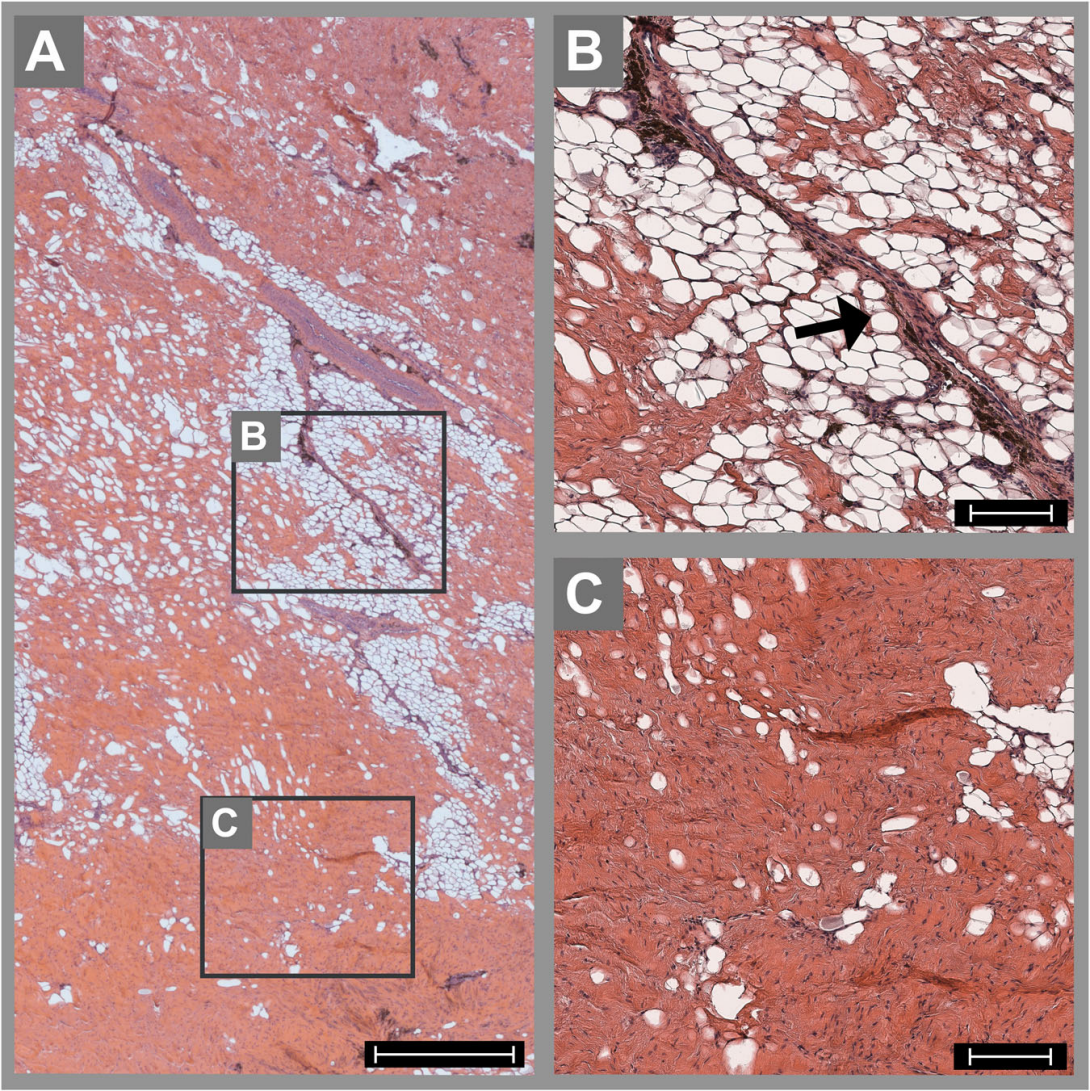
Histological specimen of the soft tissue from the forehead covering a subperiosteal NiTi implant after 16 weeks stained with hematoxylin–eosin. The tissue is mainly characterized by fat and some connective tissue (arrow in **B**) and striated muscle (**C**). No relevant signs of acute or chronic inflammation are observed. The black boxes in **A** indicate the magnified areas in **B** and **C**. The scale bar is 1000 µm in **A** and 100 µm in **B** and **C**

In some animals, the bone above the nasal cavity was thinner (due to the physiology of the animal) than the width of the intraosseous specimens. In these cases, the edge of the implants (both NiTi and titanium) loomed in the respiratory epithelium. The respiratory membrane, which was penetrated by the osteotomy preparation, had recovered completely and finally covered the implants. No signs of an inflammatory response in the membrane or the nasal cavity could be seen. The soft tissue covering the frontal bone adjacent to the implants was mainly characterized by adipocytes, connective tissue, and muscular tissue (Fig. 6). Indications of an acute inflammatory reaction, such as macrophages, granulocytes, or lymphocytes, were not evident, neither for chronic reactions, such as the appearance of plasma cells or fibrosis, nor for increased capillary ingrowth. A small number of foreign-body cells were observed in the titanium group but not in the NiTi group. However, due to the nearly normal appearance, no significant differences between the groups were detectable (Table 2).

**Table 2:** Quantification of histological signs of acute and chronic inflammation in the soft tissue adjacent to the implants

	NiTi	Titanium	Negative control	*p*-value
Macrophages	2	3	2	0.39
Granulocytes	13	12	10	0.82
Foreign-body cells	0	1	0	0.42
Lymphocytes	1	1	1	0.32
Fibrosis	0	0	0	n.a.
Capillary ingrowth	0	0	0	n.a.

Macrophages, granulocytes, foreign-body cells and lymphocytes are specified as counts per field of view and fibrosis and capillary ingrowth with a semiquantitative scale in relation to the negative control. n.a. not applicable

## Discussion

The histological specimens showed more extensive bone overgrowth at the surface of the subperiosteal NiTi implants than at the titanium implants. This seems to be a common finding, as different researchers have also described bone overgrowth on the surface of nickel–titanium implants at the cranial bone of rabbits [12, 13]. On the one hand, bone overgrowth results in additional bone–implant contact area, which is an indicator for high biocompatibility. On the other hand, titanium, as the gold-standard biocompatible implant material, resulted in less bone overgrowth. From a clinical point of view, bone overgrowth is an unintended side effect, however, the clinical relevance remains unclear, as long as bone overgrowth appears to be minimal. The occurrence of bone overgrowth indicates the high capacity of the peri-implant structures, particularly the elevated periosteum, to increase their metabolic activity and shift bone turnover toward appositional bone growth. The enrichment of the fluorescence dye in the newly formed bone suggests the periosteal response to stimulation by implant insertion. Depending on the clinical indication, anatomic site, and size of the implants, removal of the osteosynthesis material after complete bone healing might be performed routinely [14, 15]. Bone overgrowth might complicate the surgical procedure and increase the risk of intraoperative complications during material removal. Based on our findings, the 3D-printed NiTi alloy seems to be suitable for implants in cases without expected secondary-material removal. A further histological finding was the formation of slight lacunae in the bone tissue adjacent to the NiTi specimens, while mainly slight resorptions occurred in the titanium group. In general, alteration of the peri-implant tissue is an unintended side effect. However, the clinical relevance of these findings remains unclear. In our study, no loosening or dislocation of the implants was detectable. It might be plausible that in the case of thin bones, e.g., the orbital floor, the resorption and formation of lacunae can result in clinically relevant alteration of the bone. In thicker bones, slight peri-implant resorption would not limit the performance of the NiTi implants. In future studies, the effect on loadbearing and wearing on osteosynthesis material should be investigated to gain a better understanding of the clinical relevance of resorption.

In the evaluation of the bone-to-implant contact, the BIC of the NiTi plates decreased from week 8 to 16, while the BIC of titanium increased. It seems that in the long run, titanium has better biocompatibility than NiTi. However, these findings are comparable to those from other investigations. Simske et al. inserted NiTi implants into the cranium of rabbits and measured BICs of 9.2% (2 weeks), 34.9% (6 weeks) and 39.6% (12 weeks) [13]. Assad et al. determined BICs of 21.4% (3 months), 33% (6 months), and 37.6% (12 months) for NiTi implants inserted into the intervertebral bone of sheep [16]. In another study using the femur of rats, the measured BICs of NiTi implants were 51, 29, and 39% over the course of 30 weeks [17]. This last study confirms the oscillation of the BIC over time after implant insertion. As our study was terminated after 16 weeks, it would be of high interest to investigate the NiTi implants for a longer duration to see if the BIC would increase again.

One limitation of the histological probes of implants is the investigation of only thin representative slices rather than the complete adjacent tissue. However, due to radiation artifacts adjacent to solid implants of radiologically high density, three-dimensional examination, such as computed tomography (CT), cone-beam CT, or micro-CT, was not applicable in this study.

Evaluation of the peri-implant soft tissue showed a small number of inflammatory cells in both groups without any significant difference from the negative control. We conclude that these findings represent very good implant to soft-tissue biocompatibility. Postoperative clinical examination of the miniature pigs did not show any signs of allergic or inflammatory reaction. The presence of inflammatory cells is a physiological response to the surgical procedure and a well-tolerated foreign body [18, 19]. Anderson et al. confirmed this conclusion, noting that the occurrence of macrophages, foreign-body cells, and inflammatory cells is viewed as a physiological reaction toward foreign bodies such as implants and wound healing [20].

Another aspect that has to be discussed is the divergent design of the implants of the two materials. The NiTi test specimens, manufactured from a 3D-printed prototype, were compact and flat, while the titanium specimens had a mesh structure. This is a strong limitation of this study, and therefore, the conclusions have to be considered preliminary. The reason for this discrepancy is that 3D printing of the NiTi alloy is a new application that is evolving rapidly. At the time of the surgical procedure, the prototypes were the most suitable implants available. To achieve better comparability, further investigations should include NiTi specimens of an equivalent mesh design to that of the titanium implants.

## Conclusion

Nickel–titanium has great potential for numerous minimally invasive surgical applications due to its superelastic properties and reversible deformability. New fabrication methods by SLM enable the fabrication of precise patient-specific implants. In this preliminary in vivo study in miniature pigs, SLM NiTi prototypes presented sufficient in vivo biocompatibility with the bone as well as the soft tissue. The formation of lacunae seems to lack any clinical relevance but should be kept in mind in ongoing investigations. Future studies should be performed using 3D-printed implants with clinically relevant designs and with the aim of evolving the concept of minimally invasive surgery.
